# Early Surgery Does Not Seem to Be a Pivotal Criterion to Improve Prognosis in Patients with Frontal Depressed Skull Fractures

**DOI:** 10.1155/2014/879286

**Published:** 2014-08-12

**Authors:** Iuri Santana Neville, Robson Luis Amorim, Wellingson Silva Paiva, Felipe Hada Sanders, Manoel Jacobsen Teixeira, Almir Ferreira de Andrade

**Affiliations:** Division of Neurosurgery, University of São Paulo Medical School, Rua Oscar Freire 1380, 05409-010 Sao Paulo, SP, Brazil

## Abstract

*Introduction*. There has been much debate about the ideal timing of surgery of frontal depressed skull fractures (DSF). In this paper, we assess whether timing of surgery may have influenced outcome. *Methods*. Retrospective cohort of 40 consecutive patients with frontal DSF who underwent surgical treatment over a 36-month period. The patients were divided into early surgery group (ESG) which were operated within 24 h and delayed surgery group (DSG). *Results*. The population comprised 39 (97.50%) men and the mean age was 27.9 years (range, 2–81 yr). There was no difference of age (*P* = 0.53), gender male (*P* = 1.00), presence of focal lesion on head CT (*P* = 0.89), hypotension (*P* = 0.28), and hypoxia (*P* = 0.15). Mean Glasgow Coma Scale (GCS) was significantly lower in patients of ESG than DSG (8.75 and 11.7, resp., *P* = 0.02). There was no difference between the groups in relation to death (*P* = 0.13), unfavourable outcome (*P* = 0.41), late posttraumatic epilepsy (*P* = 0.64), and smell-and-taste disturbances (*P* = 1.00). Only one patient (3.5%) evolved meningitis during follow-up. *Conclusion*. We found no difference between the ESG and DSG in respect to death, unfavourable outcome, LPE, and STD.

## 1. Introduction

The presence of skull fracture on patients sustaining traumatic brain injury (TBI) is an important risk factor for intracranial lesions, such as hematomas, contusions, unfavourable outcome, and death [1–3]. Cranial fractures can be classified as depressed or linear, when they have a single trait and no displacement between the bone edges. Depressed skull fractures (DSF), one of the types of compound cranial fractures, usually resulting from blunt injuries, occur when the extent of bone displacement is greater than the full thickness of the adjacent calvarium. Compound DSF are fractures with an overlying scalp laceration and galeal disruption. Frontal DSF exhibits some peculiarities such as frequent involvement of frontal sinus and olfactory nerve and tract, which lie on the floor of the anterior cranial fossa. Frontal DSF with involvement of the inner table of the frontal bone can lead to particular complications such as central nervous system (CNS) infections in up to 15% to 30% of the patients (e.g., meningitis and brain abscess), mucocele cyst, chronic sinusitis, cerebrospinal fluid (CSF) leakage, late posttraumatic epilepsy (LPE), and smell and taste disturbances (STD) [1, 4–10]. It is worth emphasizing that CNS infections are associated with permanent neurological sequelae and other unfavourable outcomes [4, 5].

By convention, closed (nonmissile), linear cranial fractures are considered nonoperative lesions unless associated with concomitant focal lesions, such as contusions and hematomas. On the other hand, frontal DSF are treated surgically, with debridement, elevation of depressed fragments and dural repair. Operative indications may include anterior table displacement with cosmetic deformity; fractures with evidence of nasofrontal outflow obstruction; displacement or extensive commination of the posterior sinus wall, because this predicts likely dural laceration; and presence of refractory CSF leakage [6–9]. The theoretical benefits beyond cosmesis are the decrease in the incidence of infection and LPE [1, 4, 10, 11]. Opinions on the immediate handling of these patients diverge and there has been much debate on the ideal timing of surgery. Some authors advocate an immediate surgical procedure, whereas others have reported better results with delayed surgery [1, 12–19].

To describe the clinical presentation, radiological findings, and clinical outcomes as well as assess whether the timing of surgery could influence the prevalence of such outcomes, we report a case series of 40 patients with frontal DSF who underwent surgical treatment.

## 2. Clinical Material and Methods

This is a retrospective cohort of 40 consecutive patients with frontal DSF surgically treated. We included all patients admitted to the* Hospital das Clínicas, *University of Sao Paulo, from the period of January 2010 to January 2013. Frontal DSF was defined as depressed fracture involving the frontal bone including the posterior wall of frontal sinus.

The clinical data were collected from the patients' charts and included gender, age, mechanism of trauma, time between the trauma and surgery (time to surgery), admission Glasgow coma scale (GCS), presence of hypoxia, hypotension, rhinorrhea, and otorrhea on hospital admission. The head computed tomography (CT) scan images were collected from an imaging manager system Philips' iSite PACS (Philips Eletronics, USA, 2006) and interpreted by a radiologist who was blind to the patient's clinical presentation and treatment. Radiographic data, including the presence of traumatic subarachnoid hemorrhage, pneumocephalus, overlying hematoma and other associated lesions, were reviewed.

### 2.1. Clinical Outcomes

Data were collected by telephone following a structured interview. Follow-up was performed at least 3 months after the trauma and the variables evaluated were mortality, extended Glasgow Outcome Scale (eGOS), occurrence of rhinorrhea, otorrhea, meningitis, late posttraumatic epilepsy (LPE), and smell/taste disturbances (STD).

### 2.2. Dependent Variables

The primary dependent variables were death and eGOS, dichotomized as favourable outcome: 6–8 and unfavourable outcome: 1–5. Other dependent variables evaluated were LPE, meningitis, and STD.

### 2.3. Timing of Surgery

In the presence of concomitant focal lesions, we generally perform early surgery (first 24 hours). In the absence of mass lesions, the surgery is carried out as soon as the patient is stabilized and in good conditions for the procedure. In order to address timing as a critical factor to avoid complications we divided this cohort in two groups: early surgery (<24 h) and delayed surgery (>24 h).

### 2.4. Management and Surgical Technique

In our institution, patients with frontal DSF are evaluated with multislice head CT scans with 3D reconstruction. All undergo surgical treatment, especially if the posterior wall of frontal sinus is fractured. We perform a cranial approach, with bifrontal craniotomy and epidural/intradural inspection for dural lesions. We advocate the frontal sinus cranialization for all the patients. During the procedure, the fractured posterior wall of the frontal sinus is drilled away, the sinus mucosa is removed with an eletrocautery, and the nasofrontal ducts are blocked. Once dural repair is accomplished, we use a pedicle flap with pericranium and fibrin glue to ensure closure. Postoperative antibiotics (second generation cephalosporins) are used for seven days after surgery.

### 2.5. Statistical Analysis

Continuous variables were presented as mean or median and standard deviation. We considered a confidence interval of 95% and Student's* t*-test was used to make comparisons. Spearman test was used to evaluate correlations while exact Fisher test was used to make comparisons of categorical variables.

A *P* value less than or equal to 0,05 was considered to be significant. Data analyses were performed in STATA 12.0.

## 3. Results

**Figure 1:**
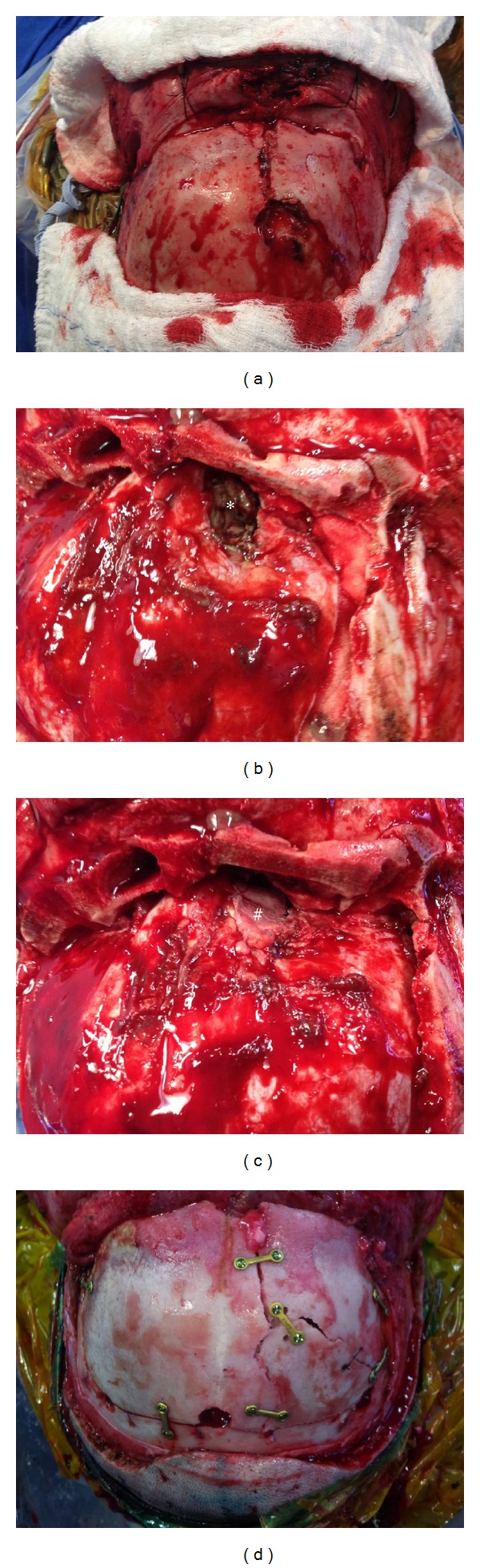
(a) Frontal depressed skull fracture exposure after skin incision; (b) ∗ dural tear is showed on epidural inspection after bifrontal craniotomy; (c) # dural repair was performed with pericranium graft; (d) final appearance after osteosynthesis and before skin closure.

During the study period, we operated approximately 900 patients for head trauma. Surgical therapy for frontal DSF was performed on 40 patients.  Figure 1 shows one of the patients treated during this study. The follow-up was completed in 28 patients (70%) and the mean follow-up period was 17.7 months (range, 4–36 months). The baseline characteristics of the patients at hospital admission are listed in Table 1. There were 39 (97.50%) men and only one (2.5%) woman. The mean age was 27.9 years (range, 2–81 yo). The main cause of injury was car accident (10 patients, 25%), followed by motorcycle/bicycle accident (10 patients, 25%). Mean GCS was 10.5 (range 3–15). The mean time to surgery was 3.3 days (range, 1–17). Rhinorrhea (6 patients, 15%), otorrhea (4 patients, 10%), hypotension (4 patients, 10%), and hypoxia (2 patients, 5%%) was present as showed. Five patients died (12.5%), all of them during hospital stay.

According to Marshall classification of diffuse traumatic brain injury [20], most of the patients were classified as grade II (24 patients, 60%) and the main focal lesion associated was brain contusion, found in 14 patients (35%), but 17 patients (42,5%) had multiple lesions. The general head CT findings are summarized in Table 2.

Table 3 shows the eGOS scores of the patients who completed follow-up. Most of the patients had complete recovery (eGOS = 8, 53.6%) and a favorable outcome was present in 71.4% (eGOS 6, 7, or 8). Age was associated with unfavourable outcome (*P* = 0.04) and death (*P* = 0.02) while LPE and STD were not associated (*P* = 0,14 and *P* = 0.84, resp.). In respect to mechanism of trauma, all the four patients who had been run over died (*P* = 0,013). eGOS (*P* = 0.088), LPE (*P* = 1.00), and STD (*P* = 0.69) were not associated with the cause of trauma. Coma and hypotension on admission were significantly associated with unfavourable outcome (*P* = 0.03 and *P* < 0.01, resp.). Also, there was a trend towards unfavourable outcome in patients with hypoxia on admission (*P* = 0.07). Hypotension and hypoxia on hospital admission was associated with death (*P* < 0.01). LPE and STD were not associated with unfavourable outcome (*P* = 1.00 and *P* = 1.00, resp.). In respect to Marshall's classification of diffuse brain injury, there was no significant association with death (*P* = 0.90) or unfavourable outcome (*P* = 0.23). Only one patient (3.5%) evolved with meningitis during follow-up.

Sixteen patients (40%) were operated within 24 hours (early surgery group, ESG).  Table 4 describes the comparison between the timings of surgery (ESG versus delayed surgery group, DSG). The groups were comparable except for the fact that mean GCS was significantly lower on patients in the ESG (8.75 and 11.7, resp. *P* = 0.02). Nevertheless, there was no difference between the groups in relation to outcomes: death (*P* = 0.13), unfavourable outcome (*P* = 0.41), LPE (*P* = 0.64), and STD (*P* = 1.00).

## 4. Discussion

In this study, we found no difference between the ESG and DSG in respect to death, unfavourable outcome, LPE, and STD. The patients had similar baseline characteristics except for the fact that the ESG had a lower GCS on admission, which was expected since the neurosurgeons tend to indicate surgery earlier on comatose patients. Additionally, only hypoxia and hypotension were predictors of death in this population. Therefore, this study provides additional knowledge for the proper management of frontal DSF.

Several neurotrauma centers advocate an immediate surgical procedure, whereas others urges for delayed surgery, reporting good results [1, 12–19, 21]. Early surgery has been used mainly for patients with concomitant intracranial lesions that require urgent evacuation. Therefore, the ideal timing for surgery is still a matter of great debate. There is only class III evidence that early surgery may be associated with better outcomes. Jennett and Miller, 1972, addressed this in a retrospective case series of 359 patients with compound skull fracture treated between 1956 and 1967. They observed that incidence of infection was significantly greater in patients with >48 h delay between injury and operation [4]. However, this study has some important biases that may have influenced these results: this paper was performed in pre-head CT scan era, which precluded the proper assessment of the extent of fractures. Moreover, patients operated after 48 h were grouped together with patients who did not ever receive surgical treatment and most of the infections were restricted to the wound, causing and increasing infection rates. So, no controlled data exist to support the timing of surgery. In patients with coexisting significant focal lesions, it is a consensus that these patients should undergo early treatment. However, when there are no mass lesions, we believe that early surgery may expose the patient to an additional trauma caused by the surgery during stabilization. Perhaps postponing surgery for a second moment may ensure that the patient is in better condition, especially those with history of hypoxia and hypotension on admission and no mass lesions.

The treatment of penetrating head injuries and depressed skull fracture has shown a gradual change over the past decades. A proper debridement and closure of scalp wounds and dural tears have been shown to decrease infection and mortality rate from compound skull fractures since a small case series of 3 patients reported by Cushing [22]. Frontal DSF can lead to complications such as meningitis, chronic sinusitis, mucocele cyst, CSF leakage, smell and taste disturbances, and late posttraumatic epilepsy [1, 16, 17, 21, 23–26]. All the patients included in our case series underwent surgical treatment. We believe that Frontal DSF, especially when the posterior wall of frontal sinus table is involved, should be treated with surgery regardless of the presence of clinical or radiological evidence of dural tear such as rhinorrhea and pneumocephalus. The rationale for this is because this predicts likely dural tear, with increased odds for CSF leakage and meningitis.

The presence of skull fractures has been shown to be associated with intracranial lesions, such as hematomas, contusions, pneumocephalus, and SAH. Ninety percent of the patients in this study had concomitant focal lesions, 82.5% had SAH, and 70% presented with pneumocephalus, corroborating the findings of other studies.

Head CT is an important tool that can show bone defects along the cranial base or indicate dural lesions through indirect signs, such as the presence of fluid in the sinuses or pneumocephalus. The presence of the latter has great importance in the radiological evaluation of these patients. Pneumocephalus was a very frequent finding in our series (70%). Scholsem et al. [19] have shown that intradural air significantly increases the risk of meningitis, and, therefore, pneumocephalus must be considered as being equivalent to CSF leakage [20]. It is not uncommon for the diagnosis of CSF leakage to be quite a challenge: the frequently performed orotracheal/nasotracheal intubation with mechanical ventilation and the high incidence of concomitant facial trauma may hamper the visualization of rhinorrhea within the emergency department.

During follow-up, only one patient presented meningitis. This was a male patient who underwent surgery on the second day of hospitalization (DSG). We were not able to perform additional data analysis in respect to meningitis, which could be possible with a greater number of patients.

Most of the patients had good to complete recovery on eGOS (71.6%). Scholsem et al., 2008, reported a good recovery in 84% of patients with traumatic CSF leakage. Most patients with Frontal DSF tend to have focal lesions due to the direct impact with a minor component of diffuse brain injury, which in part can explain the relatively good recovery of this population [19].

We reported an incidence of STD in 50% of our patients. STD are seldom reported in spite of their impact on social life; these sensations are of secondary importance when compared with vision, hearing, balance, and feeling, but, nevertheless, not unimportant [26]. Two case series reported a prevalence of STD as high as 38% and 50% of cases [19, 26]. Apparently, STD appear to be more related to the intensity of the head trauma than with the surgical technique used to correct frontal DSF [26].

This paper has the limitations inherent to retrospective studies. Moreover, the relatively small number of patients prevents a multivariate analysis of the data. Also, the complications of Frontal DSF may occur several years after trauma and the relatively short follow-up of our series may have underestimated this incidence. We also had a considerable amount of patients who did not complete the follow-up (30%), which may have influenced our results. In addition, measures of quality of life were not done in the follow-up, which could have brought insights into the critical role of STD in these patients life. This is also a single-center study, which may compromise the external validity and generalizability of the data. Finally, information about smell and taste was collected by the phone, which cannot be objectively addressed and rated by this means.

## 5. Conclusion

Time to surgery does not seem to be crucial in the prognosis of the victims, allowing surgical correction to be scheduled after patient's stabilization without compromising neurological recovery. Although 71.6% showed good recovery, half of these patients have STD, which may significantly compromise the quality of life.

## Figures and Tables

**Table 1:** Baseline data.

*n*	40 (100%)
Mean age (range) years	27.9 (2–81)
Male gender	39 (97.50%)
Mechanism of trauma	*n* (%)
Car accident	10 (25%)
Motor/bicycle accident	10 (25%)
Fall from height	5 (12.5%)
Physical assault	4 (10%)
Trampling	8 (20%)
Other causes	3 (7.5%)
Mean GCS (range)	10.5 (3–15)
Mean time to surgery (days, range)	3.3 (1–17)
Rhinorrhea	6 (15%)
Otorrhea	4 (10%)
Hypotension	4 (10%)
Hypoxia	2 (5%)

**Table 2:** Head CT scan findings.

	Patients, no (%)
Marshall diffuse axonal injury classification	
Type I	3 (7.5%)
Type II	24 (60%)
Type III	6 (15%)
Type IV	4 (10%)
Type V	3 (7.5%)
Type VI	0 (0%)
Pneumocephalus	28 (70%)
Subarachnoid hemorrhage	33 (82.5%)
Concomitant Focal lesions	
Epidural hematoma	5 (12.5%)
Subdural Hematoma	0 (0%)
Contusions	14 (35%)
Multiple lesions	17 (42.5%)

**Table 3:** Extended Glasgow outcome scale (eGOS) scores.

eGOS score	Significance	Patients, number (%)
8	Upper good recovery	15 (53.7%)
7	Lower good recovery	5 (17.9%)
6	Upper moderate disability	0 (0%)
5	Lower moderate disability	2 (7.1%)
4	Upper severe disability	0
3	Lower severe disability	0
2	Vegetative state	1 (3.6%)
1	Death	5 (17.9%)

Total		28 (100%)

**(a):** 

	ESG *n* = 16	DSG *n* = 24	*P*
Age (mean)	29.9	26.5	0.53
GCS (mean)	8.75	11.7	0.02
Male gender	16 (100%)	23 (95.8%)	1.00
Presence of concomitant focal lesions on head CT	15 (93.7%)	21 (87.5%)	0.89
Hypotension	3 (18.7%)	1 (4.2%)	0.28
Hypoxia	2 (12.5%)	0	0.15

**(b):** 

	ESG	DSG	*P*
Death	25%	4.2%	0.13
Unfavourable outcome	38.4%	20%	0.41
LPE	33.3%	21.43%	0.64
STD	50%	50%	1.00
